# Correction: Familial hypercholesterolaemia and coronary risk factors among patients with angiogram-proven premature coronary artery disease in an Asian cohort

**DOI:** 10.1371/journal.pone.0307980

**Published:** 2024-07-24

**Authors:** The PLOS ONE Staff

There is an error in affiliation 1 for authors Sukma Azureen Nazli, Yung-An Chua, Zaliha Ismail and Hapizah Nawawi. The correct affiliation 1 is: Institute for Pathology, Laboratory and Forensic Medicine (I-PPerForM), Universiti Teknologi MARA, Selangor, Malaysia.

The publisher apologizes for the error.
