# Supplementary material for: Familial hypercholesterolaemia and coronary risk factors among patients with angiogram-proven premature coronary artery disease in an Asian cohort
Source: PLoS One. 2022 Sep 2;17(9):e0273896. doi: 10.1371/journal.pone.0273896 (PMC9439256; doi:10.1371/journal.pone.0273896)
Supplement: Attachment — Submitted filename: Response to Reviewers.docx [file pone.0273896.s002.docx]

**Response to Reviewers**

Reviewer #1:

|  | Major critiques | Response |
| --- | --- | --- |
| 1. | This is a very unique cohort that patients with premature CAD account for more than 75% (145+174/145+174+101=76.0%) of all patients with CAD. Is this cohort representative of CAD population in your country? | We would like to clarify that only G1 and G2 are consist of PCAD individuals (145+174= 319). The rests in G3 and G4 (101+152= 253) are those without CAD/PCAD. Therefore, this study does not represent the proportions of PCAD among CAD cases in Malaysia.    We have added extra description in the following section to clarify the nature of G3 and G4.  Methodology - Study design and patient recruitment: (Page 7, green highlight, Lines 130-132). |
| 2. | Methodology, how group 4 patients were recruited was not mentioned. | Those in group 3 (G3) and 4 (G4) were recruited through community health screening programmes. The methodology was briefly mentioned previously, but now we have added a new paragraph to emphasise the point.  Methodology - Study design and patient recruitment: (Page 7, yellow highlight, Lines 126-128). |
| 3. | Table 1, data of central obesity for group 2 (n = 174) are missing, which is difficult to understand. | The data of central obesity for G2 (-FH/+PCAD) is missing because we did not take the measurements of waist circumference for those in this group due to the nature of recruitment for this particular group. Although the PCAD patients in G2 were consented for this study, the recruitment began way earlier than G1, G3 and G4, where the measurement of waist circumference was not part of the recruitment protocol.  For the overall analysis of regression (Results: Table 2) which involves all groups including G2 (n=572), we have no choice but to exclude central obesity.  However, we are still able to include central obesity as one of the analysis variables in regression analysis that involves FH patients in G1 and G3 (Results: Table 3 and Table 4).  Nevertheless, the absence of central obesity does not adversely affect the regression analysis and the final outcome of this study. |
| 4. | The patient number in Table 4 is 63, rather small, and the statistical analysis may not be appropriate. | Based on the central limit theorem (CLT), the distribution of sample means approximates a normal distribution as the sample size gets larger, regardless of the population's distribution. Sample sizes equal to or greater than 30 are often considered sufficient for the CLT to hold (Mascha et al., 2018).  Besides, all data for n=63 individuals were complete and variables from the univariate analysis with p value <0.25 were included in the multivariate analysis. For multivariate analysis, the value of p <0.05 were taken as significant.  Therefore, we are quite confident that our sample size for Table 4 is sufficient, and its statistical analysis is appropriate.  Reference:  Mascha, E. J., & Vetter, T. R. (2018). Significance, errors, power, and sample size: the blocking and tackling of statistics. *Anesthesia & Analgesia*, *126*(2), 691-698. |
|  | Other comments | Response |
| 1. | Abstract, 2nd sentences, “Subjects were divided into AP-CAD with FH (G1)…” “Subjects were divided into AP-PCAD with FH (G1)…” | Thank you very much for the comment. We have corrected the term “AP-CAD” to “AP-PCAD”.  Abstract: (Page 2, green highlight, Line 30). |

Reviewer #2:

|  | Comments | Response |
| --- | --- | --- |
|  | The authors have addressed my comments. However, I would very strongly recommend one change to the abstract to include the figures for Definite FH. Lines 34-36 should read "The prevalence of Definite, Potential and All FH among AP-PCAD patients were 6%(19/319), 16% (51/319) and 45.5% (145/319) respectively. It is very important to include upfront the figures for definite patients since these are consistent in findings in non-Asian studies and include patients overwhelmingly likely to have an FH-causing variant. | Thank you very much for the recommendation. We have added the prevalence for Definite FH as suggested.  Abstract: (Page 2, turquoise highlight, Lines 34-36). |
